# A Systematic Review of Medical Equipment Reliability Assessment in Improving the Quality of Healthcare Services

**DOI:** 10.3389/fpubh.2021.753951

**Published:** 2021-09-27

**Authors:** Aizat Hilmi Zamzam, Ahmad Khairi Abdul Wahab, Muhammad Mokhzaini Azizan, Suresh Chandra Satapathy, Khin Wee Lai, Khairunnisa Hasikin

**Affiliations:** ^1^Department of Biomedical Engineering, Faculty of Engineering, Universiti Malaya, Kuala Lumpur, Malaysia; ^2^Engineering Services Department, Ministry of Health Malaysia, Putrajaya, Malaysia; ^3^Department of Electrical and Electronic Engineering, Faculty of Engineering and Built Environment, Universiti Sains Islam Malaysia, Nilai, Malaysia; ^4^School of Computer Engineering, Kalinga Institute of Industrial Technology, Deemed to Be University, Bhubaneswar, India

**Keywords:** medical devices, biomedical equipment, performance evaluation, maintenance management, assessment, prediction

## Abstract

Medical equipment highly contributes to the effectiveness of healthcare services quality. Generally, healthcare institutions experience malfunctioning and unavailability of medical equipment that affects the healthcare services delivery to the public. The problems are frequently due to a deficiency in managing and maintaining the medical equipment condition by the responsible party. The assessment of the medical equipment condition is an important activity during the maintenance and management of the equipment life cycle to increase availability, performance, and safety. The study aimed to perform a systematic review in extracting and categorising the input parameters applied in assessing the medical equipment condition. A systematic searching was undertaken in several databases, including Web of Science, Scopus, PubMed, Science Direct, IEEE Xplore, Emerald, Springer, Medline, and Dimensions, from 2000 to 2020. The searching processes were conducted in January 2020. A total of 16 articles were included in this study by adopting Preferred Reporting Items for Systematic Review and Meta-Analyses (PRISMA). The review managed to classify eight categories of medical equipment reliability attributes, namely equipment features, function, maintenance requirement, performance, risk and safety, availability and readiness, utilisation, and cost. Applying the eight attributes extracted from computerised asset maintenance management system will assist the clinical engineers in assessing the reliability of medical equipment utilised in healthcare institution. The reliability assessment done in these eight attributes will aid clinical engineers in executing a strategic maintenance action, which can increase the equipment's availability, upkeep the performance, optimise the resources, and eventually contributes in providing effective healthcare service to the community. Finally, the recommendations for future works are presented at the end of this study.

## Introduction

The growing sophistication of medical equipment has significantly improved the individual and society's health (1). The advancement has improved survivability in the face of disease or injury and greatly enhanced patients' life quality through an improved diagnosis and therapeutic results. Managing assets and facilities is one of the significant features in ensuring the continuity of primary and support business activities in healthcare services (2). The delivery of healthcare services to the communities are significantly affected without effective management implementation (3–5). Medical equipment is a crucial asset that substantially contributes to the effectiveness and healthcare services quality enhancement (6, 7). As the medical equipment aids various services in the healthcare sector, the management representative, such as clinical engineers, must monitor and upkeep the assets by performing several maintenances works throughout the equipment life cycle (8, 9).

Maintenance management of medical equipment is crucial to ensure that a machine operates in accordance with manufacturer specifications and guarantees the patients and users safety (10). Failure of medical equipment may affect the healthcare services effectiveness and cause severe injury to the patients and harm the environment (11). Bahreini et al. (12) summarised that the affecting factors are management, resources, information bank, service, inspection, education, and quality control. Performance assessment is one of the activities that can be carried out regularly throughout the maintenance and repair phase to determine the medical equipment's actual condition.

Executing the assessment requires information concerning medical equipment features to produce the expected output. The expected output will assist healthcare management or clinical engineers in making essential decisions on maintenance management practises to enhance the reliability and availability of medical equipment. Furthermore, specific studies on assessment techniques within the South East Asia region, particularly in compliance with the Malaysian standard for managing medical equipment maintenance, are still lacking. In developing the present systematic review, the following research questions were addressed:

- What are the significant parameters required on the medical equipment to be applied for the reliability assessment from the previous studies?
- How do these parameters applicable to the Malaysian standard practises for managing the maintenance of medical equipment?

Selecting the significant parameters to be considered for medical equipment reliability assessment is very crucial in ensuring optimum healthcare services. In this study, the identification of these significant parameters can be applied for various types of medical equipment utilised in any healthcare institutions. In addition, we provide the review on feasibility of prediction of medical equipment reliability analysis using artificial intelligence (AI) and/or machine learning (ML) techniques based on these parameters throughout the maintenance phase of the medical equipment's life cycle. This study also leads to the revealing of the gap and novelty. The identified parameters will contribute to the comprehensive and strategic maintenance management of medical equipment, which cover three main elements of preventive maintenance (PM), corrective maintenance (CM), and replacement plan (RP). Furthermore, the reliability assessment using these parameters may fulfil and improve the medical equipment maintenance's national standard. Based on the study undertaken, none of included studies contributed on these three aspects and correlates the parameters with the relevant standards. Hence, the study aimed to identify the significant parameters of medical equipment by undertaking a systematic review of previous studies and correlate with the Malaysian standard of medical equipment maintenance management.

## Materials and Methods

### Literature Search

The systematic literature review was performed by applying the published standard, namely PRISMA in evaluating and rigorously analysing the articles related to medical equipment assessment in the databases (13). Besides, the inclusion and exclusion processes of the relevant current studies were thoroughly performed. The examination of the included study is coded to achieve the systematic review's objective in the subject area.

### Resources

The studies related to medical equipment assessment was from two primary databases, namely Web of Science and Scopus. The databases cover more than 256 studies field, including engineering and computer science studies that may increase the comprehensiveness and qualities of the article (14, 15). According to Younger (16), several established databases should be included to enhance the possibility of achieving the relevant articles in the subject area. In this study, the selected additional databases were PubMed, Science Direct, IEEE Xplore, Emerald, Springer, Medline, and Dimensions.

### Article Selection

This stage elaborates the articles selection process. There are three steps in selecting the relevant articles, namely identification, screening, and eligibility.

#### Identification

The identification and selection of the relevant studies comprise four main stages. Firstly, the subject areas' keywords were identified. The thesaurus, encyclopaedia and past researches were referred to construct appropriate keywords. Secondly, search string algorithms were developed from the keywords in January 2020 based on the Web of Science and Scopus databases' characteristics, as illustrated in Table 1. Next, several inclusions and exclusion criteria were determined to retrieve the articles from both databases (Refer to Table 2). These criteria were set because only the latest research articles in the subject area were retrieved to minimise the possibility of irrelevant topic inclusion. Besides, only English articles were considered for easier analysis preparation. Subsequently, these two search strings were applied in the databases' advanced search. Resultantly, 183 articles from Web of Science and 505 articles from Scopus were retrieved. Similar keywords were applied in the seven other databases, where 64 articles were identified. Moreover, identification of relevant studies was carried via other methods, which are websites, organisations, and citation searching (17). By using the similar keywords, there were 98 references were identified, Thus, 852 references consist of the articles and reports were retrieved in the identification stage.

**Table 1:** The search strings for Web of Science and Scopus databases.

**Database**	**Search string**
Web of Science	TS = ((“medical equipment*” OR “medical device*” OR “biomedical equipment*”) AND (“performance” OR “reliability” OR “maintenance”) AND (“assessment” OR “predict*” OR “inspect*” OR “priorit*”))
Scopus	TITLE-ABS-KEY ((“medical equipment*” OR “medical device*” OR “biomedical equipment*”) AND (“performance” OR “reliability” OR “maintenance”) AND (“assessment” OR “predict*” OR “inspect*” OR “priorit*”))

**Table 2:** The inclusion and exclusion criteria.

**Criterion**	**Eligibility**	**Exclusion**
Literature type	Journal (research articles)	Journal (review), book series, book, chapter in a book, conference proceeding
Language	English	Non-English
Timeline	Between 2000 and 2020	< 2000
Subject area	Engineering, Computer Science, Medical Information, Operations and Management	Other than Engineering, Computer Science, Medical Information, Operations and Management

#### Screening

The 852 articles and reports were divided into two to remove duplication and exclude non-related subject areas or topics during the screening process. There were 38 and 19 repeated articles in the databases and other methods, respectively. Therefore, these duplicated articles were removed, and the remaining 716 articles and 79 reports progressed to a further screening process. Three features were properly examined during the screening process: the title, keywords, and abstract. Furthermore, several considerations were considered while examining the three features. Firstly, the general terms of medical equipment or medical device or other specific equipment categorised under these general terms were mentioned in the title and keywords. Secondly, an indication of the quantitative method in assessing the medical equipment performance was depicted in the abstract. Consequently, only 85 articles and 21 reports were selected to progress to the following step.

#### Eligibility

**Figure 1:**
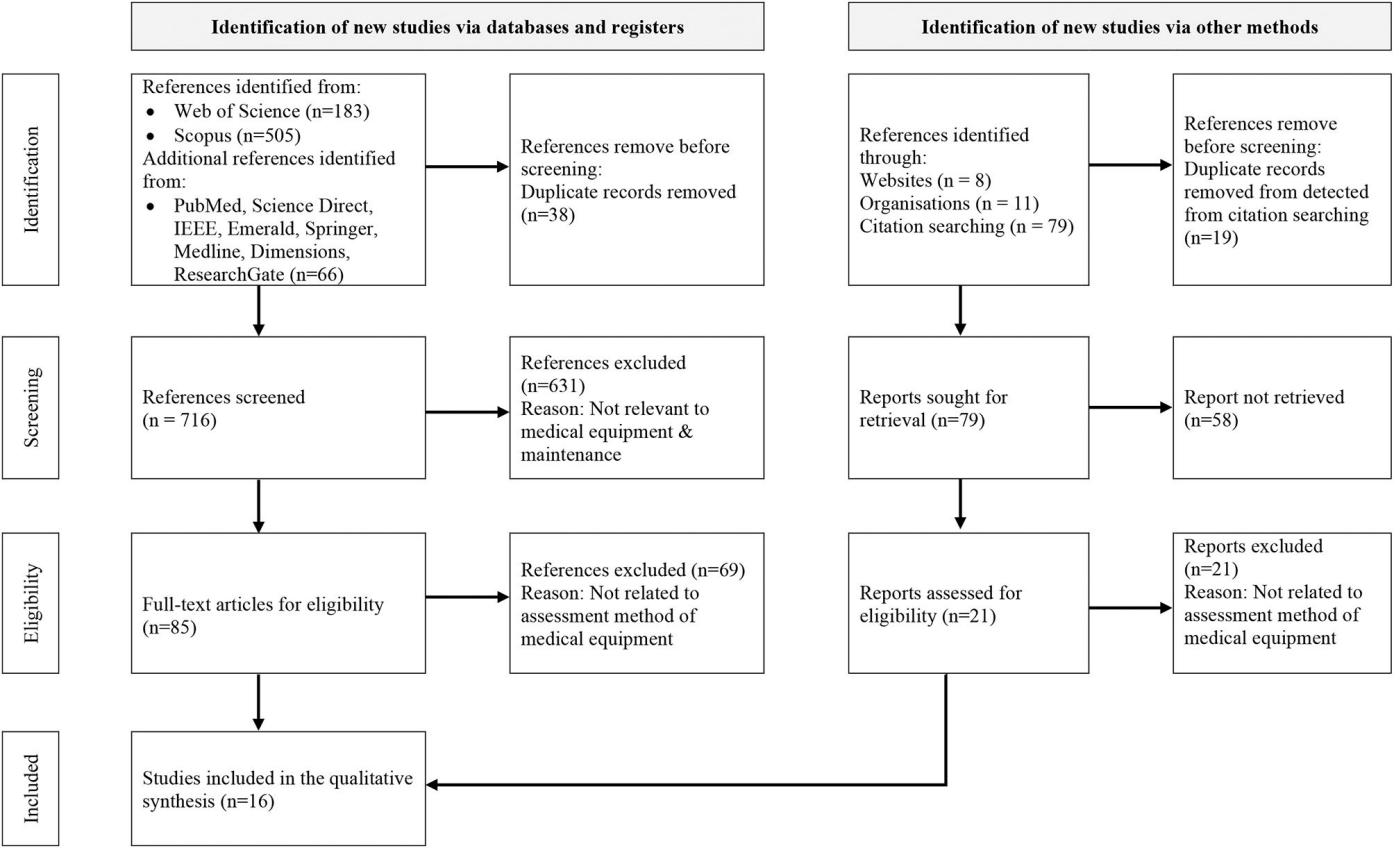
PRISMA flow chart of the study adapted from Page et al. (17).

This step involved reviewing the articles' full text to ensure that the 85 research articles and 21 reports were eligible to be synthesised and analysed. The articles' significant contents were comprehensively scrutinised to confirm that the inclusion and exclusion criteria were fulfilled. Essential elements such as study aim, input parameters, methodology technique, expected output, and desired outcomes were thoroughly assessed. Subsequently, 69 articles and 21 reports were excluded due to not utilising the quantitative method to assess the medical equipment performance and not empirical studies. Besides, an additional two relevant articles were included based on hand-searching. Therefore, a total of 16 remaining articles were included in this study, as illustrated in Figure 1.

#### Quality Assessment and Data Extraction

The qualitative analysis technique was used to assess the remaining articles. The first, second, and sixth authors performed the quality assessment of selected articles. The articles were categorised into high, moderate, and low levels that reflect the aim, input parameters, methodology technique, expected output, and desired outcomes (18). The articles must reach a high level and be agreed upon by all the authors. The compilation of extracted information was carried out by the first, second, and sixth authors and synthesised in an organised table. The third, fourth, and fifth authors subsequently checked all the synthesised data. The synthesised data was categorised by applying the thematic analysis. According to the Active Medical Device Maintenance Management developed by the Department of Standard, Malaysia, the established categories were correlated with the features. The result of input features categorisation was prudently discussed among authors. Any discrepancies or inconsistencies were resolved by consensus and until reaching reviewers agreement.

## Results

### Overall Background and Studies Findings

The analysis was carried out on 16 articles included in this study, as presented in Table 3. Based on this table, we concluded that none of the studies performed comprehensive analyses which include PM, CM, and RP. The selected studies included either one of the medical equipment reliability assessments of PM, CM, RP, and/or combination of either type of assessment. All articles were reviewed, and the motivations of each study were analysed and extracted. The articles' common traits were identified, and potential research gaps were determined. Currently, no proper protocol and early intervention exercise in assessing the performance of medical equipment were reported. The healthcare organisation faces difficulty in implementing effective maintenance management of medical equipment without proper methodological procedure and planning. Thus, the medical equipment is unable to correctly operate and could be harmful to patients and users.

**Table 3:** Authors, maintenance activity, output indicator, and outcomes.

**Authors (Region)**	**Maintenance activity**			**Output indicator**	**Outcomes**
	**PM**	**CM**	**RP**		
Kovacevic et al. (19) (Bosnia and Herzegovina)	✓	✓		Prediction accuracy.	Performance prediction, potential breakdowns, and maintenance cost optimization of infant incubators.
Badnjevic et al. (20) (Bosnia and Herzegovina)	✓	✓		Prediction accuracy.	Performance prediction, potential breakdowns, and maintenance cost optimization of defibrillators.
Saleh et al. (21) (Italy)	✓			Priority score.	Prioritisation of highly critical medical equipment.
Hernandez-Lopez et al. (22) (Mexico)	✓			Preventive maintenance index value.	Lifespan maximisation, operations cost optimization, and prioritisation of medical equipment.
Jamshidi et al. (23) (Canada)	✓	✓		Priority score.	Availability enhancement, cost optimisation, and prioritisation of medical equipment.
Faisal et al. (24) (Egypt)			✓	Replacement score.	Availability enhancement, cost optimisation, and prioritisation of medical equipment.
Tawfik et al. (25) (Egypt)	✓	✓		Risk classification score.	Cost optimisation and prioritisation of medical equipment.
Jarikji et al. (26) (Lebanon)			✓	Replacement score.	Prioritisation of highly critical medical equipment.
Aridi et al. (27) (Lebanon)			✓	Replacement score.	Cost optimisation and prioritisation of medical equipment.
Hamdi et al. (28) (Jordan)	✓	✓		Priority score.	Reliability and availability improvement, and prioritisation of medical equipment.
Hutagalung et al. (29) (Indonesia)	✓	✓		Criticality score.	Availability enhancement and cost minimisation through effective maintenance plan.
Taghipour et al. (30) (Canada)	✓	✓		Criticality score.	Condition identification and prioritisation of medical equipment.
Ben Houria et al. (31) (Tunisia)	✓	✓		Criticality score.	Reliability and availability enhancement, cost optimisation, and prioritisation of high-risk medical equipment.
Oshiyama et al. (32) (Brazil)			✓	Classification division.	Prioritisation of medical equipment.
Saleh and Balestra (33) (Italy)	✓			Criticality index score.	Prioritisation of critical medical equipment.
Ismail et al. (34) (Lebanon)	✓	✓		Risk severity number.	Failures mitigation and prediction of medical equipment.

*PM, Preventive Maintenance; CM, Corrective Maintenance; RP, Replacement Plan*.

Since a large number of medical equipment and multiple functions are utilised in healthcare institutions, the equipment shall be monitored and correctly maintained to sustain performance and safety levels. However, maintenance management could be challenging if the healthcare provider encounters several problems regarding insufficient competent personnel and available resources, such as replacement parts and funds. According to the World Health Organisation (WHO), initial expenses, and operating expenditures are two categories of required financial resources in maintaining medical equipment (35). Corciova et al. (36) added that maintenance expenses represent a significant portion of the entire healthcare system which required 15–60% of the total cost to operate. Improper maintenance may affect performance and safety which greatly gave significant impact on the expenditure of healthcare institutions (37). Wu et al. (38) proved that practising effective maintenance management within 2 years improves the medical equipment availability and minimises the operating costs which exceeded one million dollars.

The computerised inventory system significantly assists healthcare management in managing equipment and maintenance activities. Applying the appropriate methodological technique in processing big data generates useful indicators that may assist clinical engineers in strategising the maintenance planning and further action course. The identification of the medical equipment criteria is essential to produce valuable indicators. Based on the analysis from the included articles, the justification for the criteria identification was referred from the previous literature, data collection and extraction, expert judgement via survey, input based on customer requirement, and adapting from international standards and national guidelines.

The relevant data were collected, processed, calculated, and analysed accordingly based on the identified criteria. Only one scientific methodological technique was involved in generating the expected output by referring to the 12 articles (19–30). Nevertheless, according to Ben Houria et al. (31), a combination of three techniques generated the expected output. The combination of two techniques was observed in the studies performed by Oshiyama et al. (32), Saleh and Balestra (33), and Ismail et al. (34), respectively. The proposed techniques were tested on the real dataset of various types of medical equipment particulars and maintenance information within a specific period.

The conclusion from the review of the 16 articles is that the healthcare institutions are capable of optimising the maintenance cost, improving the monitoring activity, managing the maintenance activities with available workforces and resources, prioritising PM and CM, assessing the equipment's actual lifespan for the purpose RP, selecting the best maintenance management strategy based on the current situation by referring to the output indicator.

### Main Findings

The outcomes from the selected articles using thematic analysis produced eight categories of significant parameters in assessing the medical equipment condition and reliability. The application of thematic analysis was carried out to develop the appropriate themes of medical equipment's parameters. Based on the thematic analysis, these parameters are found to be significant for the AI/ML network as the input parameters. The initial stage of theme development procedures was the compilation of each parameter extracted from the 16 selected articles. In this phase, the categories of input parameters were properly analysed to extract the description used in the selected articles to address the research gaps. Then, in the second phase, various terms of input parameters were converted into general category via the themes, applications, or ideas classification. From the analysis, we found out that there were many terms used in previous studies, however, several of them can be addressed under the same group. Eventually, the thematic analysis has generated a total of eight categories of input parameters, which described the characteristics of medical equipment utilised in healthcare institutions.

The observation was also carried out according to the studies outcomes. There were seven categories of input parameters extracted from the selected articles as tabulated in Table 4. In general, the outcome was to classify the medical equipment in accordance with maintenance management activities. The medical equipment maintenance activities comprise of PM, CM, and RP. The prioritisation was made based on the medical equipment characteristics toward the strategic maintenance management activity.

**Table 4:** Categories of input parameters.

**Category**	**Description**	**Outcomes**
Equipment features	Device age Technology age Manufacturer Type Service support Equipment complexity	Prioritise equipment maintenance Prioritise preventive maintenance Prioritise replacement plan Prioritise equipment and maintenance strategy selection
Function	Service intention	Classify equipment risk Prioritise corrective maintenance Prioritise equipment and maintenance strategy selection Prioritise equipment maintenance Prioritise preventive maintenance Prioritise replacement plan
Maintenance requirement	Performance and safety test Inspection Calibration Maintenance Complexity checking Frequency Maintenance time	Classify equipment risk Frequency of preventive maintenance Prioritise corrective maintenance Prioritise equipment and maintenance strategy selection Prioritise equipment maintenance Prioritise preventive maintenance Prioritise replacement plan
Performance	Efficiency Failure Number of corrective maintenances Downtime Useful life Service life	Frequency of preventive maintenance Prioritise corrective maintenance Prioritise equipment and maintenance strategy selection Prioritise equipment maintenance Prioritise preventive maintenance Prioritise replacement plan
Risk and safety	Dangers Failure probability, consequence, and detectability Recalls and hazards alerts Safety and environment Patient and staff safety	Classify equipment risk Prioritise equipment and maintenance strategy selection Prioritise equipment maintenance Prioritise preventive maintenance Prioritise replacement plan
Availability and Readiness	Alternative and backup Clinical acceptability Device and service criticality	Classify equipment risk Prioritise corrective maintenance Prioritise equipment and maintenance strategy selection Prioritise equipment maintenance Prioritise replacement plan Prioritise preventive maintenance
Utilisation	Operations Location and environment	Classify equipment risk Prioritise equipment and maintenance strategy selection Prioritise equipment maintenance Prioritise preventive maintenance Prioritise replacement plan Prioritise corrective maintenance
Cost	Maintenance cost Environmental factors	Prioritise replacement plan Prioritise equipment maintenance Prioritise equipment and maintenance strategy selection Prioritise corrective maintenance

The generated eight categories of input parameters will assist the clinical engineers to perform the medical equipment reliability assessment. Understanding of the equipment reliability may lead to proper maintenance activity, which subsequently increase the availability of medical equipment with optimised resources. The measurement of these parameters using AI/ML techniques will comprehensively enhance the monitoring of medical equipment performance and utilisation status through predictive maintenance model. This predictive model is able to mitigate the potential failures, deterioration, and obsolescence. Findings from the selected articles produced eight categories of the significant input parameters in assessing the medical equipment condition, as tabulated in Table 4.

#### Equipment Features

Equipment features comprise several characteristics designed for the equipment and exist since the unit is manufactured. One of the parameters used to assess the medical equipment condition is age (19, 20, 22–24, 26, 27, 29–31). The equipment age reflects the overall condition of the equipment. This is because, the equipment is typically performed well at an early age, and fewer failures are observed. However, as the age increases, the equipment starts to degrade. In another studies conducted by Badnjevic et al. (20) and Kovacevic et al. (19), manufacturer's name and equipment's modality are another two parameters that were included in their reliability assessment. Prior to that, Faisal et al. (24), included the service availability as one of the input parameters in assessing the equipment condition for the RP prioritisation. The service availability of the medical equipment includes warranty, documentation, training, and compatible spare part. Meanwhile, studies by Saleh et al. (21) and Saleh and Balestra (33) also considered equipment complexity as the input parameter in their medical device reliability assessment.

#### Function

The function of the equipment here reflects on the intended use of the equipment in healthcare services delivery. The equipment function is divided into several forms of services namely life support, therapeutic, diagnostic, analytical, and miscellaneous.

#### Maintenance Requirement

Maintenance requirements involve activities and tasks to ensure that the medical equipment is sustained in the expected condition in terms of functionality and physical. The complexity of carrying out the maintenance procedure is different among the equipment types. The maintenance, servicing, or restoration of this equipment requires a skilled person to dismantle and replace the replacement or faulty part. The procedure is also time-consuming. Thus, the equipment can be unavailable for an extended period if the performance of maintenance activity is carried out ineffectively.

#### Performance

The reliability of the medical equipment highly depended on the performance that can be measured from the efficiency and uptime. The equipment effectiveness can be observed from the usage and service life. The performance of medical equipment should be vitally monitored and ensured as described by the manufacturer. The excellent performance of medical equipment can mitigate the interruption of healthcare services to the public.

#### Risk and Safety

In delivering the healthcare services such as diagnosis and treatment, patients and clinicians must be kept safe without exposure to any hazard that may cause severe injury. The risk and safety of the equipment can be predicted by studying the failure aspects (23, 28, 30). The authority may issue the recalls and hazards alert if any incident occurs involving medical equipment utilisation by instructing the user to immediately stop using the equipment to prevent further possible danger to clinicians and patients (23, 30). In addition, hazards may when mishandling, misdiagnosis, inappropriate treatment or error are made by the operator (30, 31). Hazards may also exist from the operation or physical of the medical equipment (21, 25, 33).

#### Availability and Readiness

The availability and readiness of medical equipment are vital to ensure that healthcare services to patients can be delivered without compromise. The category consists of the alternatives and backup units, device criticality, and user acceptability. The breakdown of equipment is inevitable due to normal wear and tear, or ageing may interfere with the effectiveness of healthcare services. Moreover, the importance of healthcare services will cause equipment to become a critical necessity due to unavoidable circumstances (25–27, 29–31). Thus, alternative units must be ready for critical times (24, 25, 27–31).

#### Location

The assessment of the medical equipment condition can also be undertaken by observing the unit utilisation level. The utilisation level can be affected by the location of equipment and the situation where the equipment is used to deliver healthcare services to the public (21, 22, 26, 33). According to the authors, the frequency of medical equipment usage and turning to be essential depending on the type and activity of healthcare services provided to patients, such as anaesthetising, operating theatres, and others. Furthermore, the equipment may be extensively or rarely used depending on the healthcare services situation.

#### Cost

Cost is one of the crucial aspects in managing medical equipment maintenance and replacement activities. According to the studies performed by Ben Houria et al., reducing the cost of operations involving medical equipment is crucial. The operational costs must be below the allocated budget (31). Findings from Faisal et al. (24) reported that maintenance costs should not be over 25% of the procured medical equipment cost over the past 3 years. In other study conducted by Oshiyama et al. (32) suggested that the CM cost should be within 3 to 5% of the medical equipment purchased price. According to Hutagalung et al. (29), maintenance costs can be reduced by enhancing the availability of medical equipment through effective maintenance management. With regards to the previous findings stated before, the maintenance cost highly reflected on the reliability of the medical equipment. The frequent failures do not only lead to the excessive maintenance tasks and disruption to the healthcare services, but also involve extra expenses. When the equipment requires high maintenance operation and the imposed cost reached to the specific limits, the equipment is no longer reliable and fit for utilisation. This condition is known as beyond economic repair.

## Discussion

### Overall Findings

Previous studies demonstrated the importance of assessing medical equipment in planning for necessary action within healthcare institutions. The first important consideration in developing the medical equipment performance assessment is determining the appropriate input parameters (12). However, no single technique can be applied to all the input parameters. The selection of input parameters must be appropriate and applicable to the expected output. According to Mahfoud et al. (39), the outcome of the medical equipment assessment associates with the maintenance strategies. The availability of an existing dataset comprising medical equipment details and maintenance history is one of the factors in selecting the appropriate input parameters. The difference in input parameters applied can be processed to generate similar output.

The second consideration is the optimum processing technique based on the myriad of medical equipment data. As mentioned earlier, many scientific methods were developed which can be used to compute the input data and eventually generate an expected output for assessment purposes (40). However, the ML technique application is observed to be a better technique compared to the conventional techniques. This is due to the capability of the ML algorithm in testing the predictive high output accuracy by applying the accurate and significant input data.

The results obtained from the studies made by Badnjevic et al. (20) and Kovacevic et al. (19) showed that the generated output achieved above 89% accuracy where Random Forest and Decision Tree reached around 99% of accuracy in predicting both selected medical equipment performance. Therefore, both authors concluded that improved supervision, quality and safety in managing medical equipment maintenance could be achieved which eventually optimised the cost of maintenance. However, the ML techniques utilised in both studies were developed based on only one type of medical equipment. Consideration of applying to various types of medical equipment would be more practical to be utilised in healthcare facility management. This is because, various types of medical equipment have difference functionality and required specific assessment to ensure their reliability to be used in healthcare services.

The third consideration in developing the medical equipment condition assessment technique is to determine the expected output. One of the indications in identifying the expected output is observing the trend of the occurred problems (41). From the list of observations, the trend is translated into a specific objective to resolve the problem. The review concludes that the clinical engineers faced several common issues, such as the unavailability of medical equipment due to malfunctioning, insufficient workforces (i.e., competent technical staff), and limited resources (i.e., limited resources budget). Effective maintenance management must be established to overcome these problems and prevent severe consequences. The prioritisation by assessing the existing medical equipment condition can be undertaken while working within the current workforce and resources.

### The Medical Equipment Reliability Assessment From the Malaysian Perspective

The Malaysian government spent approximately RM27 million in 2018 for new procurement and upgrading initiatives of medical equipment in public healthcare facilities to provide efficient healthcare services to the public (42). The Malaysian government also executed a new leasing programme involving six main medical equipment for 5 years starting from 2019, comprising a maintenance scheme with an approximate cost of RM19.7 million.

In the private sector, KPJ Healthcare Berhad, a leading private healthcare service in Malaysia with more than 20 hospitals throughout the country, procured medical equipment worth around RM136 million in 2019, showing an increment of 32% from the previous year (43). The evidence from both sectors indicates that massive investment in procurement and maintenance of medical equipment is necessary in delivering effective healthcare services to the community. Therefore, the efficient maintenance management of medical equipment during operations is vital to maximise the life span of the unit and ensure the investment is worthy.

During the eleventh Malaysia Plan, there was a need of highly technological medical equipment to meet the essentials for various kind of diseases advanced management (44). These critical machines such as computerised tomography (CT) and magnetic resonance imaging (MRI) were required in facilitating the medical practitioners for detecting, diagnosing, and treating the critical diseases. The National Medical Devices Survey was conducted in both public and private healthcare sectors and found out that the ratio of MRI number and population was two per million, whereas CT was 4 per million. This finding indicated the lower ratios than of Organisation for Economic Co-operation and Development (OECD) countries.

Referring to the Ministry of Health report for three consecutive years starting from 2017, the percentages of hospitals and public health facilities outpatient attendees had slightly increased in average of 2.64%, whereas the hospitals day care attendees had significantly escalated to 15.8% (45–47). Furthermore, the hospitals admissions had marginally increased to 5.96%. This indication drove the Malaysian government to expand the secondary and tertiary care services. From the year of 2015–2018, the number of hospital beds was increased by 3.3%, in which the increment of 11% applied to intensive care units (48). Although the improvement was made, the existing ratio of 1.9 beds to every 1,000 Malaysia populations is still below than the initial target. From the initiative programme implementation, the Malaysian government provides 67% of total beds in the country. Other expansion programmes that initiated by the Malaysian government were the development a new replacement hospital in east-coast region, the extension of the new complexes in the existing hospitals, and the new hospital in central region. These development activities require the expansion of facility, equipment, manpower, and services.

Apart from this expansion initiative, the medical-based day care services such as paediatric, oncology, and haematology require specific medical equipment for chemotherapy, blood transfusion, and haemodialysis (49). Therefore, the Ministry of Health Malaysia has come out with a general policy to enhance the availability of medical equipment for strengthen the healthcare services in the country. Besides, based on the inspection carried out by the internal audit of Ministry of Health Malaysia has recommended that the improvement of medical equipment is required to facilitate the healthcare services to the public (50).

The application of medical equipment in facilitating the healthcare services is crucial. The Malaysian government provided a huge amount of funds in procuring and maintaining the medical equipment in the country. The procurement of medical equipment includes for a new development of hospitals and replacement of the disposed units due to beyond economic repair (42).

Subsequently, this sub-section discusses the correlation on eight categories of input parameters as set by the Malaysian standard, namely the Code of Practise for Good Engineering Maintenance Management of Active Medical Devices (MS 2058:2018) (51). The primary function of this body is to encourage and promote global competitiveness through reliable standardisation and accreditation services governed by the Standards of Malaysia Act 1996 Act 549 (52). Several normative references comprising other interconnected MS 2058:2018 guidelines and national acts are available to develop a comprehensive standard and comply with the legislative requirement.

One crucial related act, specifically for managing the medical equipment, is the Medical Device Act 2012 (53). This act is intended to provide statutory regulation for medical devices in Malaysia and shall be complied with by all relevant parties, such as authorised representatives, manufacturers, and service providers. This act is also under the enforcement of the Malaysian governmental regulatory body, namely the Medical Device Authority, based on the establishment of the Medical Device Authority Act 2012 (54). Act 737 regulates the entire life cycle of medical equipment covering three main phases, namely pre-market, placing in market and post-market.

According to Annex P of MS 2058:2018, ten factors are proposed to assess the medical equipment for the RP. Based on the observation and comparison made with the included studies as shown in Table 5, these factors can also be used as an input to assess the medical equipment condition for maintenance prioritisation.

**Table 5:** Comparison between factors proposed in MS 2058:2018 and included studies.

**Replacement factor**	**Included studies factor**
Asset condition	Failure detectability
Asset status	Performance
Asset usage	Mission criticality; operational impact; utilisation
Frequency of breakdowns	No. of corrective maintenance, frequency of failures, rate of failures
Asset age	Device age
Obsolescence	Support availability; technology age; vendor support
Safety alert	Risk; failure consequences; recalls and hazard alerts
Maintenance cost	Cost of corrective maintenance
Availability of back up equipment	Alternative availability
User recommendation	Clinical acceptability

Firstly, according to the observation of the factors proposed in the MS 2058:2018, the asset age is directly similar to the first category grouped based on the analysis in this study, namely the equipment features. Furthermore, most authors highly utilise equipment age to obtain an indication of prioritising maintenance and RP. The second factor that is similar to the equipment feature category was obsolescence. This factor is quite identical to service support because if there is no service support in the market, the restoration work involves replacement parts or any maintenance services for related equipment can be delivered. Therefore, the factors of asset age and obsolescence are similar to equipment features.

The results of comparisons between all the factors proposed in the MS 2058:2018 with two of the eight categories, namely function and maintenance requirement, found no similarity. Next, the performance category comprises several parameters involving efficiency, failure, downtime, uptime, and the number of corrective maintenances performed. In comparison with the MS 2058:2018, the performance category seems equivalent to the frequency of breakdown where the medical equipment performance can be measured by assessing the failure rate. Furthermore, factors such as asset status and asset condition seem to be related to this category.

The next category initiated in this study was risk and safety. This category is crucial to mitigate any potential hazards posed to the patient and clinician. From the comparison made, the factor of safety alert proposed in MS 2058:2018 correlated with the risk and safety category. The correlation was due to the risk and safety category involving the recalls and hazards alerts that can be declared or issued by the local authority body (53), manufacturer or locally authorised representative. The availability and readiness were in a category consisting of the element of correlation between equipment and service criticality. In MS 2058:2018, the factors that were observed as most similar were the availability of backup equipment and user recommendation. These factors demonstrate the criticality in ensuring equipment availability in assisting the healthcare services at the clinically acceptable level.

Asset usage is one of the 10 factors proposed in MS 2058:2018 that indicates the extensive utilisation level of the medical equipment. Direct similarity with the category of utilisation was apparent when compared against the categories in this study. A direct similarity can be identified between the maintenance cost factor proposed in MS 2058:2018 and the cost categorised in this study. Table 6 tabulates the correlation between the eight categories summarised based on the review in this study with the 10 factors proposed in MS 2058:2018.

**Table 6:** Correlation between study categories and factors in MS2058:2018.

**Category^**a**^**	**Malaysia standard^**b**^**
Equipment features	Asset age Obsolescence
Function	None
Maintenance requirement	None
Performance	Frequency of breakdown Uptime Asset status Asset condition
Risk and safety	Safety alert
Availability and readiness	Availability of backup equipment User recommendation
Utilisation	Asset usage
Cost	Maintenance cost

a *Categories are based on the review of included studies*.

b *Factors are based on MS 2058:2018*.

The national standard of MS 2058:2018 helps the clinical engineers significantly in managing the maintenance of medical equipment in Malaysia. There are several vital relevant acts and standard were referred for the development and compilation of this national standard. It covers various types of equipment in all maintenance stages starting from equipment acceptance to disposal process. This standard also includes main and sub-element of maintenance activities, which are PM, CM, and RP. The ultimate purpose is to ensure a proper maintenance is performed for continuous performance of medical equipment.

The adherence of MS 2058:2018 may optimise the performance of medical equipment utilised in healthcare institution. The optimised performance of medical equipment reduces error while delivering the healthcare services to public. Any difference in measurement could give bad impact on the healthcare services especially to the patients. It is important to ensure that the equipment can give accurate and precise measurement during therapeutic, diagnostic, and analytic procedures. The reliability of healthcare services really depends on effectiveness of medical equipment. The optimised equipment does not only assist during healthcare procedures, but improve the availability of the equipment while needed. Moreover, it also upkeeps in terms of the safety aspect from any possible failure, which may cause severe harmful against the users and patients. Thus, the optimization of medical equipment performance may upkeep the level of healthcare services and reduce the cost of operations.

## Conclusion

The current study on the medical equipment assessment indicated a fundamental understanding of how the assessment contributes toward the effectiveness in delivering healthcare services to the community. The ultimate contribution from this study is that the healthcare institutions are capable of providing better healthcare services to the public by the medical equipment availability, upkeep the safety level by avoiding any failures of equipment that may cause hazards, and prepare and allocate sufficient budget on the expenditure of equipment maintenance and replacement activities.

The review summarised that the assessment of medical equipment conditions will assist the clinical engineers to increase the availability of the equipment in healthcare institutions. Furthermore, the equipment availability will assist the clinical engineering department in healthcare institutions to achieve medical equipment maintenance management effectiveness by prioritising the activity according to the urgency. This prioritising approach may eventually optimise the operational cost and work with available resources.

According to the comparison of the findings in this study with MS2058:2018, the categories classified based on the input parameters extracted from the previous studies and have been correlated with proposed factors in the national standard. However, improvement can be made by adding several factors that covered the function and maintenance requirement categories. The proposal for selecting the input parameters depends on clinical engineers' required objective to overcome the current issue experienced in healthcare institutions. The selected parameters can be practical with the support of accurate existing data.

The expected beneficial output indicated from the assessment technique depends on the experimental input parameters. The output can be generated through systematic processes and professional ways rather than the perception by adapting the appropriate methodological technique. This output can assist clinical engineers in making a proper decision to take the right action to overcome the current issue. Therefore, this study provides recommendations that will be useful for future research:

1) Development of a comprehensive strategic medical equipment maintenance management that covers three main activities, which are PM, CM, and RP. The system shall prioritise the medical equipment at each maintenance activity by measuring the criterion of input parameters proposed in this study. In addition, considering these three activities will provide a comprehensive assessment to the healthcare providers in prioritising resources and proposing appropriate solution in timely manner.
2) Applying ML techniques in assessing the medical equipment condition and reliability. The predictive nature of ML will provide active action in healthcare industry in anticipating medical equipment's failures. Current solutions are based on passive actions which greatly impacted healthcare services providers. Thus, the advancements of ML techniques are deemed to be a practical solution in medical equipment predictive maintenance in mitigating severe failures, optimising resources, improving availability, and upkeeping performance.
3) Imposing adaptive framework on medical equipment reliability based on the functionality of healthcare providers. The capability of ML algorithms in predicting prioritisation of medical equipment maintenance will enable accurate and precise assessment based on the contributing factors of the medical equipment. The framework will be adaptive to the nature of the healthcare institutions' function since the predictive nature of ML algorithm able to find patterns and trends based on the previous scenarios. Thus, specific model can be easily adopted by healthcare providers in ensuring optimised services to community.

Therefore, by applying appropriate techniques that drive the compliance of national standards and statutory requirements, the review provided a new insight in adopting AI and/or ML algorithms which can be aligned in any government's standard in providing better healthcare services.

## Author Contributions

AZ and KH designed and developed the study protocol as well as major contributors to the article writing. AZ, AA, and KH performed the identification, screening, eligibility, quality assessment, and information extraction of the articles. MA, SS, and KL checked all the synthesised data and approved the final version to be submitted for publication. All authors have substantial contributed to the article.

## Funding

This work was supported by the University Malaya Research Grant Faculty Programme (RF010-2018A) and International Funding from Motorola Solution Foundation (IF014-2019).

## Conflict of Interest

The authors declare that the research was conducted in the absence of any commercial or financial relationships that could be construed as a potential conflict of interest.

## Publisher's Note

All claims expressed in this article are solely those of the authors and do not necessarily represent those of their affiliated organizations, or those of the publisher, the editors and the reviewers. Any product that may be evaluated in this article, or claim that may be made by its manufacturer, is not guaranteed or endorsed by the publisher.

## Abbreviations

AI: Artificial Intelligence
CM: Corrective Maintenance
ML: Machine Learning
MS2058:2018: Code of Practise for Good Engineering Maintenance Management of Active Medical Devices
PM: Preventive Maintenance
PRISMA: Preferred Reporting Items for Systematic Review and Meta-Analyses
RP: Replacement Programme
WHO: World Health Organisation.
